# Feature-enhanced iTransformer: A two-stage framework for high-accuracy long-horizon traffic flow forecasting

**DOI:** 10.1371/journal.pone.0340389

**Published:** 2026-01-09

**Authors:** Yonghui Duan, Yucong Zhang, Xiang Wang, Yuan Xue, Zirong Wang, Di Wu

**Affiliations:** 1 Department of Civil Engineering, Henan University of Technology, Zhengzhou, Henan, China; 2 Department of Civil Engineering, Zhengzhou University of Aeronautics, Zhengzhou, Henan, China; National University of Defense Technology, CHINA

## Abstract

Accurate and reliable long-horizon traffic flow prediction is a cornerstone of modern Intelligent Transportation Systems (ITS), yet it remains challenging due to the complex, non-linear, and dynamic spatio-temporal dependencies inherent in traffic data. While recent Transformer-based models have shown promise, they are typically end-to-end systems that couple feature extraction and sequence prediction, which can limit their ability to fully leverage multi-faceted domain information. To address this, we propose a two-stage framework, the Feature-Enhanced iTransformer (FE-iTransformer), founded on an extract-and-enhance philosophy. The framework first employs a comprehensive Feature Enhancement Module (FEM) to distill a global context vector from spatio-temporal dynamics, periodic patterns, and temporal context—without relying on a predefined graph structure. Subsequently, an innovative per-step feature enhancement mechanism uses this global vector to enrich the original input sequence, yielding an information-rich representation that is then processed by a strong iTransformer backbone for final prediction. The effectiveness of FE-iTransformer is validated through extensive experiments: ablation studies on two classic datasets (Freeway and Urban) provide compelling evidence for the efficacy of the two-stage design, demonstrating that introducing FEM significantly improves the pure iTransformer backbone; supplementary experiments on the large-scale PEMS08 benchmark further confirm scalability and long-horizon performance, reducing Mean Absolute Error (MAE) by 19.1% over the vanilla backbone in the 120-minute forecasting task. Importantly, this study targets no-graph/weak-graph settings and does not aim to surpass graph-prior models; rather, it offers a deployment-ready, graph-free alternative when the roadway graph is unavailable or unreliable.

## 1. Introduction

The rapid pace of global urbanization has led to unprecedented challenges in urban mobility, with traffic congestion emerging as a critical bottleneck affecting economic productivity, environmental quality, and public safety [1,2]. Intelligent Transportation Systems (ITS) have become a cornerstone of modern smart city initiatives, leveraging advanced information and communication technologies to monitor, analyze, and manage complex traffic networks [3,4]. At the heart of ITS lies the task of accurate and reliable traffic flow prediction, which provides the foundational data for a myriad of applications, including dynamic traffic signal control [5], proactive congestion mitigation [6], and intelligent route guidance [7]. By anticipating future traffic states, transportation authorities can optimize network efficiency, reduce travel times, and enhance overall road safety, making traffic flow prediction are search area of paramount importance [8].

Despite these advancements, achieving high-precision traffic flow prediction is an inherently challenging task due to the complex and dynamic nature of traffic data. Several core technical challenges must be addressed. First, traffic flow exhibits intricate spatio-temporal dependencies [9]. The traffic state at a specific location is not only determined by its own historical patterns (temporal dependency) but is also strongly influenced by the conditions of upstream, downstream, and even functionally similar but geographically distant road segments (spatial dependency) [10]. Second, traffic systems are characterized by high non-linearity and dynamics, frequently affected by non-recurrent events such as traffic accidents, extreme weather, and public holidays, which can cause abrupt changes in flow patterns [11]. Third, traffic data is embedded with multiple periodic patterns, such as daily rush hours and weekly variations between weekdays and weekends, which are crucial for long-term forecasting [12].

To address these challenges, methods have evolved from statistical models to deep learning. Early approaches, including statistical models like ARIMA and machine learning methods like Support Vector Regression (SVR), often struggle to capture the complex non-linear relationships inherent in traffic data [2]. In recent years, deep learning models have become the dominant paradigm. Methods based on Recurrent Neural Networks (RNNs), such as Long Short-Term Memory (LSTM), excel at modeling temporal sequences but often fall short in effectively capturing long-range dependencies and complex spatial correlations [13,14]. Convolutional Neural Networks (CNNs) capture local spatial features but are limited by fixed receptive fields, hindering their adaptability to non-Euclidean road networks [15]. Graph Convolutional Networks (GCNs) have shown great promise by explicitly modeling the topological structure of road networks [16]. However, their performance heavily relies on a pre-defined, often static, adjacency matrix, which may not capture the dynamic and time-varying nature of spatial dependencies in real-world traffic [17,18].

More recently, Transformer-based models have demonstrated remarkable success in capturing long-range dependencies in sequential data [19]. However, standard Transformers, when directly applied to traffic forecasting, may overlook the local context and sequential nature of time series data. Furthermore, their quadratic computational complexity with respect to sequence length remains a significant challenge for long-term prediction tasks [20]. This has spurred research into more efficient and specialized Transformer variants, yet a comprehensive solution that effectively integrates multi-faceted traffic characteristics without relying on a predefined graph structure remains an open challenge.

To address the aforementioned limitations, we propose a novel framework, the Feature-Enhanced iTransformer (FE-iTransformer). Our approach is founded on a two-stage “extract-and-enhance” philosophy. First, a graph-free Feature Enhancement Module (FEM) distills a global context vector from spatio-temporal, periodic, and temporal data. This vector then enriches the input sequence via a per-step mechanism. Finally, an iTransformer backbone processes the enhanced sequence for forecasting. This design effectively decouples complex feature extraction from long-range dependency modeling.

The main contributions of this paper are summarized as follows:

1. We propose a novel two-stage framework, named Feature-Enhanced iTransformer (FE-iTransformer), for high-accuracy traffic flow forecasting, which decouples the complex task into distinct stages of feature enhancement and enhanced prediction.
2. We design a comprehensive Feature Enhancement Module (FEM) that effectively captures multi-faceted traffic characteristics by concurrently modeling spatio-temporal dynamics, periodic patterns, and temporal context without relying on a predefined graph structure.
3. We introduce an innovative per-step feature enhancement mechanism that leverages the global context vector distilled by the FEM to enrich the original input sequence, significantly improving the model’s performance, especially in long-horizon forecasting scenarios.
4. Extensive experiments demonstrate the effectiveness and robustness of our approach. In-depth ablation studies on our primary datasets (Freeway and Urban) rigorously validate the efficacy of each component of our design, while supplementary experiments on the large-scale PEMS08 benchmark confirm the framework’s superior performance and scalability in a challenging, real-world forecasting environment.

This work targets no-graph or weak-graph prior settings that frequently arise in practice. We therefore study a graph-free two-stage pipeline in which multi-source temporal context is extracted and injected before variable-wise attention. We do not claim superiority over graph-prior models under graph-rich conditions; rather, we provide a deployment-ready alternative when constructing or maintaining a reliable adjacency is costly or impractical. In line with this scope, our empirical comparisons focus on non-graph baselines (linear, recurrent, and transformer families). Effectiveness is demonstrated by vertical comparisons against the iTransformer backbone and horizontal comparisons against representative non-graph baselines, with long-horizon settings (up to 120-min) emphasized. Supplementary results on PEMS08 corroborate scalability.

The remainder of this paper is organized as follows. Section 2 reviews the related work. Section 3 details the methodology of our proposed model. Section 4 presents the experimental results and analysis. Finally, Section 5 concludes the paper.

## 2. Related work

The methodologies for traffic flow prediction have evolved substantially, moving from classical statistical models to complex deep learning frameworks that can better navigate the intricate dynamics of modern transportation systems [2,21]. Early approaches were dominated by statistical methods like Auto-Regressive Integrated Moving Average (ARIMA), which models time series based on their historical patterns [22], and machine learning techniques such as Support Vector Regression (SVR), which leverages kernel methods to handle non-linearities [23]. While foundational, these models often struggle to capture the highly complex and non-linear spatio-temporal dependencies inherent in traffic data without extensive manual feature engineering [24].

The rise of deep learning introduced a new era for traffic forecasting. Recurrent Neural Networks (RNNs) and their advanced variants, notably the Long Short-Term Memory (LSTM) network introduced by Hochreiter & Schmidhuber [25], became the primary tools for modeling temporal sequences. Their ability to capture long-term dependencies made them a popular choice in various hybrid frameworks, such as the work by Wang et al.[14], which combined LSTM with Bayesian Neural Networks for uncertainty quantification. However, a core limitation of RNNs is their inherent inability to directly model spatial relationships. To address this, researchers began incorporating Convolutional Neural Networks (CNNs). For instance, Zhang et al.[26] demonstrated a CNN-based approach that treated spatio-temporal data as a 2D matrix. While effective for extracting local spatial patterns, the fixed receptive fields of CNNs limit their capacity to model the non-Euclidean topology of road networks and dynamic long-range spatial correlations, a challenge that recent hybrid models like CCNN-former from Liu et al.[27] and the convolutional transformer by Sattarzadeh et al.[28] aim to resolve by combining CNNs with Transformer architectures. Beyond these established architectures, recent advancements have introduced novel paradigms to address data scarcity and modeling complexity. For instance, generative adversarial networks [29] and diffusion models [30], along with virtual-real fusion simulations [31], have been effectively employed to address data scarcity and synthesize high-fidelity representations. Furthermore, emerging architectures like Kolmogorov-Arnold Networks [32] have demonstrated superior capability in capturing intricate non-linear dynamics compared to traditional MLPs.

To explicitly model the graph structure of road networks, Graph Neural Networks (GNNs) emerged as a milestone technology. The seminal work of Yu et al.[33] on Spatio-Temporal Graph Convolutional Networks (STGCN) provided a powerful framework for simultaneously capturing spatial and temporal features. Building on this, the field has rapidly advanced. Chen et al.[16] incorporated attention mechanisms into dynamic GCNs to improve traffic speed prediction, while Mu et al.[9] developed a hierarchical GNN to capture multi-scale spatio-temporal semantics. Similarly, Ji et al.[10] proposed a hybrid framework combining depthwise separable convolutions with GNNs for efficient multi-scale feature extraction. Despite their success, a fundamental challenge for most GNN-based models remains their reliance on a pre-defined adjacency matrix. As pointed out by Diao et al.[17] and Zheng et al.[18], this static graph often fails to represent the dynamic nature of traffic correlations, leading to research on adaptive and dynamic graph generation techniques. Concurrently, some non-graph spatiotemporal optimization approaches have also emerged to address these limitations. For instance, Chen et al.[34] utilized simulation-based optimization for sensor placement, and Hu et al.[35] developed multi-branch spatiotemporal attention networks for predictive maintenance. These studies demonstrate the potential of capturing complex dependencies and optimizing structural configurations without relying on pre-defined static graphs.

Specifically for sequence modeling tasks such as traffic flow forecasting, the Transformer architecture [36] has demonstrated exceptional capability with its self-attention mechanism, offering an unparalleled ability to capture long-range dependencies. This has led to a surge of Transformer-based models in traffic forecasting. To address the computational complexity of the original Transformer for long sequences, Wu et al.[37] proposed Autoformer, which uses an Auto-Correlation mechanism. More recently, Nie et al.[38] developed PatchTST, which improves efficiency and performance by segmenting time series into patches. A significant innovation came from Liu et al.[39] with iTransformer, which inverts the attention mechanism to operate on the variable (sensor) dimension, a design particularly effective for multivariate time series and which serves as the backbone for our model. While these models achieve state-of-the-art results, they typically function as end-to-end systems, coupling feature extraction and prediction.

Through this review of existing literature, a clear research gap emerges: the lack of a framework that decouples multi-faceted feature extraction from long-range dependency modeling without relying on predefined graph structures. To fill this gap, we propose the Feature-Enhanced iTransformer (FE-iTransformer), a novel two-stage architecture designed to first extract a rich, global context representation and then use it to enhance the input sequence for a more robust and accurate prediction.

## 3. Methodology

This section elaborates on the technical architecture of our proposed model, the Feature-Enhanced iTransformer (FE-iTransformer). The detailed structure of each component will be presented in the following subsections.

### 3.1. Problem definition

The task of traffic flow forecasting aims to predict a sequence of future traffic states for a set of N spatially distributed sensors, given their historical observations. The historical traffic flow data is represented as a tensor $𝐗\in {\text{R}}^{B\times P\times N}$, where B is the batch size, P is the length of the historical input sequence (e.g., 32 time steps), and N is the number of sensors. In this work, we focus on a univariate forecasting setting where each sensor provides a single feature (traffic flow).

To enhance long-horizon prediction accuracy, our model also utilizes two types of auxiliary information:

1. Periodic Data (${𝐏}_{h}$): A tensor ${𝐏}_{h}\in {\text{R}}^{B\times P\times C_{per}}$, which captures historical daily and weekly patterns. Here, $C_{per}=\text{2}$ represents the two periodic sequences.
2. Time Features (𝐓): A tensor $𝐓\in {\text{R}}^{B\times P\times C_{time}}$ which encodes contextual information from timestamps (e.g., hour of the day, day of the week). Here, $C_{time}=\text{4}$.

Given the historical observations 𝐗 and the auxiliary inputs (${𝐏}_{h},𝐓$), the objective is to predict the traffic flow for all N ensors over the next Q time steps. The target sequence is denoted as $𝐘\in {\text{R}}^{B\times Q\times N}$. The entire forecasting task can thus be formulated as learning a mapping function F, parameterized by Θ, which maps the historical information to the future traffic sequence:


$$𝐘=F\left(𝐗,{𝐏}_{h},𝐓;\Theta \right)$$ (1)

The goal is to optimize the parameters Θ by minimizing the discrepancy between the predicted sequence $\hat{𝐘}$ and the ground truth sequence 𝐘.

### 3.2. Overall architecture

**Fig 1:**
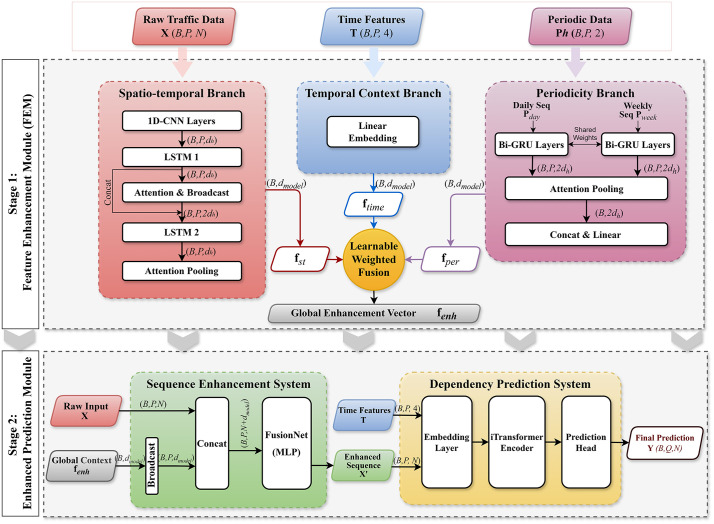
Overall structure of the FE-iTransformer model.

To tackle the challenges of long-horizon traffic forecasting, we propose the Feature-Enhanced iTransformer (FE-iTransformer), a novel framework designed with a clear, two-module architecture founded on an “extract-and-enhance” philosophy. The overall architecture is illustrated in Fig 1.

The model is composed of two primary modules: the Feature Enhancement Module (FEM) and the Enhanced Prediction Module. The workflow begins with the raw traffic data 𝐗, periodic data ${𝐏}_{h}$, and time features 𝐓 being fed into the FEM. This module processes the inputs through three parallel branches—a Spatio-temporal Branch, a Periodicity Branch, and a Temporal Context Branch—to distill a single, comprehensive Global Enhancement Feature Vector, denoted as ${𝐟}_{enh}$. Subsequently, this vector is passed to the Enhanced Prediction Module, where it is used to enrich the original input sequence 𝐗 at each time step. Finally, this information-rich sequence is processed by a powerful iTransformer predictor to generate the final forecast. The detailed design of each component will be presented in the following sections.

### 3.3. Feature enhancement module (FEM)

The Feature Enhancement Module (FEM) serves as the feature extraction engine of the first stage. Its objective is to distill a compact, high-dimensional global context vector ${𝐟}_{enh}\in {\text{R}}^{d_{model}}$ from the heterogeneous input data, including the historical traffic flow 𝐗, periodic sequences ${𝐏}_{h}$, and temporal timestamps 𝐓. To achieve this, the FEM employs a multi-branch architecture consisting of three parallel components.

#### 3.3.1. Spatio-temporal branch.

To capture both local spatial correlations and global temporal dynamics from the raw traffic sequence $𝐗\in {\text{R}}^{B\times P\times N}$, we design a hierarchical “Summarize-Broadcast-Reprocess” mechanism. This branch processes the input through four sequential steps:

Step 1: Local Feature Extraction. We first employ a 1D Convolutional Neural Network (CNN) to extract local spatial features. Treating the sensor dimension N as channels, the convolution operation transforms the raw input into a latent feature sequence ${𝐗}_{cnn}$:


$$\begin{array}{c} {𝐗}_{cnn}=\sigma \left(\text{Conv1d}\left(𝐗\right)\right)\in {\text{R}}^{B\times P\times d_{h}}, \end{array}$$ (2)

where σ denotes the activation function and $d_{h}$ is the hidden dimension within the branch.

Step 2: Initial Sequential Encoding & Summarization. The feature sequence ${𝐗}_{cnn}$ is fed into a primary Long Short-Term Memory (LSTM) network to capture temporal dependencies, yielding a hidden state sequence ${𝐇}_{1}\in {\text{R}}^{B\times P\times d_{h}}$. To distill the global context, we apply an attention-based pooling layer:


$$\begin{array}{c} {𝐇}_{1}={\text{LSTM}}_{\text{1}}\left({𝐗}_{cnn}\right), \end{array}$$ (3)

$$\begin{array}{c} \alpha_{t}=\text{Softmax}\left({𝐯}^{𝐓}\text{tanh}\left({𝐖}_{a}{𝐇}_{1,\;\;t}\right)\right), \end{array}$$ (4)

$$\begin{array}{c} {𝐜}_{global}=\sum_{t=\text{1}}^{P}\alpha_{t}{𝐇}_{1,t}, \end{array}$$ (5)

where ${𝐜}_{global}\in {\text{R}}^{d_{h}}$ serves as a summarized context vector representing the holistic state of the current observation window. ${𝐖}_{a}$ and 𝐯 are learnable parameters.

Step 3: Broadcast and Reprocess. A critical innovation of our design is the Broadcast-Reprocess mechanism. The global context ${𝐜}_{global}$ is broadcast (repeated) across the temporal dimension to form a sequence ${𝐂}_{broadcast}=\{{𝐜}_{global},\ldots ,{𝐜}_{global}\}$ of length P. This sequence is concatenated with the initial hidden states ${𝐇}_{1}$ and fed into a secondary LSTM. This allows the model to re-examine the local temporal features under the guidance of the global context:


$$\begin{array}{c} {𝐇}_{2}={\text{LSTM}}_{\text{2}}\left(\text{Concat}\left({𝐇}_{1},{𝐂}_{broadcast}\right)\right). \end{array}$$ (6)

Step 4: Final Aggregation. Finally, the refined sequence ${𝐇}_{2}$ is aggregated via another attention pooling layer to produce the final spatio-temporal feature vector ${𝐟}_{st}$:


$$\begin{array}{c} {𝐟}_{st}=\text{AttentionPooling}\left({𝐇}_{2}\right)\in {\text{R}}^{d_{model}}. \end{array}$$ (7)

#### 3.3.2. Periodicity branch.

Traffic flow exhibits strong cyclical characteristics, most notably daily and weekly periodicities. To capture these recurring patterns while strictly adhering to temporal causality, this branch processes the periodic input tensor ${𝐏}_{h}$. Strictly implementing a Historical Value Retrieval strategy to prevent data leakage, we construct ${𝐏}_{h}$ by retrieving specific past observations relative to the prediction start time t: the daily sequence ${𝐏}_{day}$ is retrieved from ${𝐗}_{t-S_{day}}$ (i.e., exactly 24 hours ago), and the weekly sequence ${𝐏}_{week}$ is from ${𝐗}_{t-\text{7}\times S_{day}}$ (i.e., exactly 7 days ago).

Shared-Weight Processing. Assuming that traffic evolution patterns are consistent across different time scales, we employ a weight-sharing strategy. Both ${𝐏}_{day}$ and ${𝐏}_{week}$ are processed independently by the same Bidirectional Gated Recurrent Unit (Bi-GRU) network with shared parameters $\Theta_{gru}$:


$$\begin{array}{c} {𝐇}_{day}=\text{Bi}-\text{GRU}\left({𝐏}_{day};\Theta_{gru}\right), \end{array}$$ (8)

$$\begin{array}{c} {𝐇}_{week}=\text{Bi}-\text{GRU}\left({𝐏}_{week};\Theta_{gru}\right). \end{array}$$ (9)

The resulting hidden sequences are compressed into context vectors ${𝐟}_{day}$ and ${𝐟}_{week}$ via attention pooling. These are then concatenated and projected to form the final periodic feature vector ${𝐟}_{per}$:


$$\begin{array}{c} {𝐟}_{per}=\text{Linear}\left(\text{Concat}\left({𝐟}_{day},{𝐟}_{week}\right)\right)\in {\text{R}}^{d_{model}}. \end{array}$$ (10)

#### 3.3.3. Temporal context branch.

This branch encodes explicit timestamp information from the tensor 𝐓 (e.g., hour of day). Since the immediate future is most strongly correlated with the current time context, we isolate the feature vector ${𝐭}_{last}$ corresponding to the final time step of the input window. This vector is mapped to the latent space via a Multi-Layer Perceptron (MLP):


$$\begin{array}{c} {𝐟}_{time}=\text{MLP}\left({𝐭}_{last}\right)\in {\text{R}}^{d_{model}}. \end{array}$$ (11)

#### 3.3.4. Learnable weighted fusion.

To adaptively integrate the features from the three branches, we employ a learnable weighted fusion mechanism. Let $𝐰\in {\text{R}}^{\text{3}}$ be a learnable parameter vector. The fusion weights β are obtained via a Softmax function:


$$\begin{array}{c} \beta =\text{Softmax}\left(𝐰\right)=\left[\beta_{st},\beta_{per},\beta_{time}\right]. \end{array}$$ (12)

The final global enhancement vector $f_{enh}$ is computed as the weighted sum:


$$\begin{array}{c} {𝐟}_{enh}=\beta_{st}{𝐟}_{st}+\beta_{per}{𝐟}_{per}+\beta_{time}{𝐟}_{time}. \end{array}$$ (13)

This vector ${𝐟}_{enh}$ encapsulates the comprehensive multi-source information and is subsequently used to enrich the input sequence in the Enhanced Prediction Module.

### 3.4. Enhanced prediction module

The Enhanced Prediction Module constitutes the second stage of the FE-iTransformer framework. Its primary function is to leverage the rich, contextual information encapsulated in the Global Enhancement Feature Vector (${𝐟}_{enh}$) to refine the original input sequence before making the final forecast. This module consists of two main components: a per-step feature enhancement mechanism and the iTransformer predictor.

#### 3.4.1. Per-step feature enhancement.

This mechanism represents a core innovation of our architecture, designed to inject the distilled global context into each individual time step of the input sequence. The process unfolds as follows: First, the Global Enhancement Feature Vector ${𝐟}_{enh}\in {\text{R}}^{d_{model}}$ is projected by a linear layer to align its dimensionality with the sensor space, resulting in a projected vector ${𝐟}_{enh}^{{}^{\prime }}$. Then, for each time step t in the historical input sequence 𝐗, the corresponding feature vector ${𝐱}_{t}\in {\text{R}}^{N}$ is concatenated with the entire projected global vector ${𝐟}_{enh}^{{}^{\prime }}$. This combined vector is then passed through a dedicated Fusion Network, which is a multi-layer perceptron (MLP), to produce the enhanced feature vector for that time step, ${𝐱}_{t}^{{}^{\prime }}$:


$$\begin{array}{c} {𝐱}_{t}^{{}^{\prime }}=\text{FusionNet}\left(\text{Concat}\left({𝐱}_{t},{𝐟}_{enh}^{{}^{\prime }}\right)\right). \end{array}$$ (14)

By repeating this process for all P time steps, we construct a new, information-rich sequence, the Enhanced Sequence ${𝐗}^{{}^{\prime }}=\left({𝐱}_{\text{1}}^{{}^{\prime }},{𝐱}_{\text{2}}^{{}^{\prime }},\ldots ,{𝐱}_{\text{P}}^{{}^{\prime }}\right)\in {\text{R}}^{B\times P\times N}$. Each element in this new sequence is now explicitly aware of the global spatio-temporal context summarized by the FEM.

The adoption of a concatenation-based MLP for feature fusion, as opposed to elementary operations like element-wise addition, is a deliberate design choice to enhance representational capacity. While linear summation assumes a simple superposition of context and input, traffic dynamics often involve complex, non-linear dependencies between global trends and local states. The MLP architecture facilitates high-order feature interactions, allowing the model to adaptively recalibrate the importance of the global context vector relative to the local input at each time step. This capability ensures that the injected context serves as a dynamic reference rather than a static bias, thereby maximizing the information gain for the subsequent prediction backbone.

#### 3.4.2. iTransformer predictor.

The final prediction is generated by a powerful iTransformer backbone [3 9], which is highly effective for long-sequence forecasting. The enhanced sequence ${𝐗}^{{}^{\prime }}$ serves as the primary input to the iTransformer backbone.

The input to the iTransformer encoder is formed by combining a value embedding of ${𝐗}^{{}^{\prime }}$ with a temporal embedding derived from the original time features 𝐓. The core of the predictor is a multi-layer iTransformer encoder. As proposed in the original work, its self-attention mechanism critically operates across the variable (sensor) dimension rather than the temporal dimension, allowing it to effectively capture dependencies among the N sensors across the entire sequence. The output from the encoder is then passed to a final linear projection layer, which acts as the prediction head, to produce the final forecast $\hat{𝐘}\in {\text{R}}^{B\times Q\times N}$.

### 3.5. Training objective

The parameters Θ of the FE-iTransformer model are optimized by minimizing a hybrid loss function that combines the strengths of two widely used regression metrics: the Mean Absolute Error (MAE) and the Root Mean Squared Error (RMSE). This composite loss leverages the robustness of MAE to potential outliers in traffic data, while also benefiting from the sensitivity of RMSE to large prediction errors.

The final loss function, L, is a weighted sum of these two components, controlled by a hyperparameter α. In our experiments, missing values in the ground truth are masked and ignored during the calculation of both metrics. The overall objective is to minimize the following function:


$$\begin{array}{c} L=\alpha \cdot L_{MAE}+(\text{1}-\alpha )\cdot L_{RMSE}, \end{array}$$ (15)

where α is set to 0.7 to place a slightly greater emphasis on the MAE component, thereby promoting a more stable training process.

## 4. Experiments

To systematically evaluate the performance of the proposed FE-iTransformer model, we conduct a series of comprehensive experiments. This chapter is organized to present a multi-faceted validation of our framework. First, we detail the experimental settings, including the datasets, evaluation metrics, and baseline models. Second, we present our main results and in-depth analyses on our primary datasets (Freeway and Urban), where we establish the model’s state-of-the-art performance and conduct rigorous ablation studies to validate our core design principles. Third, we address the crucial question of scalability by presenting supplementary experiments on the large-scale PEMS08 benchmark. Finally, we provide qualitative analysis through visualizations to offer more intuitive insights into the model’s predictive behavior.

### 4.1. Experimental settings

This section details the comprehensive experimental setup designed to rigorously evaluate our proposed FE-iTransformer framework. We describe the datasets used for validation, the metrics for performance evaluation, the state-of-the-art baseline models for comparison, and the specific implementation details to ensure the reproducibility of our results.

#### 4.1.1. Datasets.

Our empirical evaluation is conducted on three real-world benchmark datasets, carefully selected to validate our proposed framework from different perspectives, and all datasets are sourced from the Performance Measurement System (PeMS). Our primary experiments are conducted on two classic datasets, Freeway and Urban, whose moderate scale and distinct traffic characteristics provide an ideal environment for detailed model analysis and rigorous ablation studies. To further validate the scalability and generalization of our findings in a more complex setting, we also include a supplementary evaluation on the large-scale PEMS08 benchmark.

Freeway Dataset: This dataset contains traffic flow data collected from 7 sensors deployed on the SR99-S freeway in District 10, California. It is characterized by high-speed and relatively structured traffic patterns, serving as a standard benchmark for highway traffic prediction. In our work, we utilize data spanning from September 18th, 2017, to March 4th, 2018.

Urban Dataset: This dataset comprises traffic flow data from 7 sensors located on urban streets in District 4, Oakland, California. It represents a more complex traffic environment with frequent stops, intersections, and diverse traffic patterns, posing a greater challenge for forecasting models. The time range of the data used is consistent with the Freeway dataset.

PEMS08 Dataset: This large-scale dataset was collected from 170 sensors on the highways of the San Bernardino area over 62 days, from July 1st to August 31st, 2016. Its larger and more complex road network topology makes it a challenging benchmark for testing a model’s ability to handle intricate, large-scale spatio-temporal dependencies, and thus serves as a robust testbed for the scalability of our framework.

The datasets are split strictly chronologically into training (64%), validation (16%), and testing (20%) sets to maintain temporal causality. The exact date boundaries are provided in **Table 1**. Furthermore, regarding the construction of periodic features at the boundaries (e.g., the beginning of the test set), we retrieve the necessary historical data from the preceding validation or training sets. For the initial samples of the entire dataset where historical data is unavailable (indices t < 24h or t < 7d), we apply **zero-padding** to ensure that no future data or invalid negative indices are accessed. This strategy guarantees a rigorous leak-free evaluation.

**Table 1:** Statistics and chronological partitioning boundaries of the experimental datasets.

Dataset	Sensors	Time Range	Subset	Date Boundary (Start – End)	Samples
Freeway	7	Sep 2017- Mar 2018	Train (64%)	2017/09/18–2018/01/03	30966
			Val (16%)	2018/01/03–2018/01/30	7741
			Test (20%)	2018/01/30–2018/03/04	9677
Urban	7	Sep 2017- Mar 2018	Train (64%)	2017/09/18–2018/01/03	30966
			Val (16%)	2018/01/03–2018/01/30	7741
			Test (20%)	2018/01/30–2018/03/04	9677
PEMS08	170	Jul 2016- Aug 2016	Train (64%)	2016/07/01–2016/08/09	11,420
			Val (16%)	2016/08/10–2016/08/19	2,855
			Test (20%)	2016/08/20–2016/08/31	3,569

Note: All datasets are collected at a 5-minute sampling interval. To prevent data leakage, the datasets are split strictly chronologically. The exact date boundaries ensure that the testing phase occurs strictly after the training and validation phases.

#### 4.1.2. Evaluation metrics.

The performance of our proposed model and the baseline methods is quantitatively assessed from different perspectives using three widely-adopted evaluation metrics. Mean Absolute Error (MAE), Root Mean Squared Error (RMSE), and Mean Absolute Percentage Error (MAPE)—measure the magnitude of prediction errors.

Mean Absolute Error (MAE): This metric measures the average magnitude of the errors in a set of predictions, without considering their direction. It is generally robust to outliers. The formula is defined as:


$$\begin{array}{c} MAE=\frac{\text{1}}{M}\sum_{i,j,k\in M}\left|{\hat{y}}_{ijk}-y_{ijk}\right|, \end{array}$$ (16)

where M is the set of observed (non-missing) data points, ${\hat{y}}_{ijk}$ is the predicted value, and $y_{ijk}$ is the ground truth value.

Root Mean Squared Error (RMSE): This metric is the square root of the average of squared differences between prediction and actual observation. It is more sensitive to large errors than MAE, penalizing them more heavily. The formula is:


$$\begin{array}{c} RMSE=\sqrt{\frac{\text{1}}{M}\sum_{i,j,k\in M}{\left({\hat{y}}_{ijk}-y_{ijk}\right)}^{\text{2}}}. \end{array}$$ (17)

Mean Absolute Percentage Error (MAPE): This metric expresses the prediction error as a percentage of the actual value, providing a relative and easily interpretable measure of accuracy. The formula is:


$$\begin{array}{c} MAPE=\frac{\text{1}\text{0}\text{0}\%}{M}\sum_{i,j,k\in M}\left|\frac{{\hat{y}}_{ijk}-y_{ijk}}{y_{ijk}}\right|. \end{array}$$ (18)

#### 4.1.3. Baseline methods.

To comprehensively evaluate the performance of our proposed FE-iTransformer, we compare it against a diverse and representative set of baseline methods. These baselines range from classical machine learning models to state-of-the-art deep learning architectures, providing a rigorous benchmark for our approach. The selected methods are as follows:

SVR (Support Vector Regression): A classical machine learning model that utilizes kernel methods to effectively capture non-linear relationships in the data.

LSTM (Long Short-Term Memory): A seminal recurrent neural network (RNN) architecture specifically designed to capture long-term temporal dependencies in sequential data.

DLinear: A simple yet surprisingly effective model for long-term forecasting, which is based on a single linear layer and has been shown to outperform many complex models.

Transformer: The original Transformer architecture adapted for time series forecasting, serving as a fundamental benchmark for attention-based models.

Autoformer: An efficient Transformer variant designed for long-sequence forecasting, which introduces a decomposition architecture and an Auto-Correlation mechanism.

PatchTST: A state-of-the-art Transformer-based model that segments time series into patches, enabling the model to capture local semantic information more effectively.

iTransformer: A recent state-of-the-art model that inverts the role of time steps and variables in the Transformer’s attention mechanism. As our FE-iTransformer utilizes iTransformer as its predictor backbone, this serves as the most crucial baseline to directly quantify the performance gain brought by our proposed Feature Enhancement Module (FEM).

#### 4.1.4. Implementation details.

For a fair comparison, all models were implemented in PyTorch and trained using the Adam optimizer with an initial learning rate of 0.0005 and a weight decay of 1e-4. We employed a learning rate scheduler that reduces the learning rate on a plateau of the validation loss. The models were trained for a maximum of 100 epochs with a batch size of 32. An early stopping strategy was applied with a patience of 15 epochs to prevent overfitting and select the best model based on its performance on the validation set.

The historical input sequence length (P) was set to 32 time steps. The prediction horizon (Q) was set to 4 steps, corresponding to forecasts at future intervals of 30, 60, 90, and 120 minutes. For our proposed FE-iTransformer and all other Transformer-based baselines, the model dimension ($d_{model}$) was set to 512, the number of attention heads ($n_{heads}$) was set to 8, and the dropout rate was 0.1. Specifically for our FE-iTransformer, the number of encoder layers ($e_{layers}$) was set to 3.

### 4.2. Main results and analysis on primary datasets

This section details the core experimental results on our primary datasets, Freeway and Urban. The analysis is structured to first establish the overall superiority of the FE-iTransformer against a comprehensive set of baselines, and then to conduct in-depth ablation studies to attribute this performance gain to our proposed architectural innovations.

#### 4.2.1. Comprehensive performance comparison.

The comprehensive quantitative results for all models on the Freeway and Urban datasets are presented in Table 2. The performance is evaluated at four future prediction horizons: 30, 60, 90, and 120 minutes. For each horizon, we report the MAE, RMSE, MAPE, and R² metrics. The best-performing model for each metric is highlighted in bold.

**Table 2:** Comprehensive performance comparison on freeway and urban datasets.

Time	Models	Metric-Freeway			Metric-Urban		
MAE	RMSE	MAPE	MAE	RMSE	MAPE
30min	LSTM	11.84	16.06	18.43%	19.06	26.46	15.35%
	SVR	11.62	15.84	17.25%	17.28	23.96	13.57%
	DLinear	13.58	17.98	22.25%	18.52	25.82	14.22%
	Transformer	11.63	15.90	16.50%	**16.17**	22.98	**11.71%**
	iTransformer	11.43	15.73	16.43%	16.37	23.16	12.15%
	Autoformer	11.47	15.82	16.77%	16.60	23.86	12.95%
	PatchTST	11.66	15.98	17.40%	17.14	23.92	12.97%
	FE- iTransformer	**11.19**	**15.33**	**15.92%**	16.23	**22.92**	11.79%
60min	LSTM	13.02	17.60	20.54%	20.33	28.17	16.15%
	SVR	12.98	17.61	19.65%	20.20	27.82	16.44%
	DLinear	12.22	16.43	18.41%	17.77	24.96	13.30%
	Transformer	12.51	17.11	17.66%	17.24	24.58	12.64%
	iTransformer	12.68	17.44	18.60%	18.30	25.86	13.99%
	Autoformer	12.91	17.80	19.25%	18.31	26.71	14.64%
	PatchTST	12.55	17.35	17.99%	17.82	24.69	13.24%
	FE- iTransformer	**11.40**	**15.54**	**16.18%**	**16.64**	**23.59**	**12.09%**
90min	LSTM	14.44	19.47	23.06%	22.09	30.49	17.86%
	SVR	14.49	19.56	22.35%	22.88	31.55	18.96%
	DLinear	12.55	16.76	19.82%	18.23	25.67	13.45%
	Transformer	13.41	18.32	18.87%	18.50	26.69	13.58%
	iTransformer	14.08	19.36	21.13%	19.92	28.25	15.71%
	Autoformer	14.71	19.66	22.34%	20.46	28.63	16.27%
	PatchTST	14.34	19.45	21.95%	19.75	27.22	15.29%
	FE- iTransformer	**11.61**	**15.77**	**16.64%**	**17.10**	**24.36**	**12.39%**
120min	LSTM	15.99	21.54	26.00%	24.17	33.38	20.11%
	SVR	15.92	21.34	25.08%	25.18	34.76	20.96%
	DLinear	14.92	19.46	25.50%	20.53	28.59	15.90%
	Transformer	14.22	19.61	20.23%	19.72	28.53	14.56%
	iTransformer	15.57	21.30	24.01%	21.58	30.49	17.54%
	Autoformer	15.12	20.11	23.88%	21.03	30.07	17.26%
	PatchTST	15.59	21.73	24.97%	21.70	31.13	18.15%
	FE- iTransformer	**11.86**	**16.11**	**17.29%**	**17.52**	**25.03**	**12.70%**

Second, the comparison with contemporary Transformer-based architectures reveals a more nuanced but equally important superiority. While models like Transformer, Autoformer, and PatchTST possess powerful sequence modeling capabilities, our FE-iTransformer consistently demonstrates a clear edge. This suggests that simply applying a powerful backbone is insufficient. The key differentiator lies in our two-stage “extract-and-enhance” paradigm. By first distilling a potent, globally-aware context vector and then using it to enrich the input sequence, our framework provides the predictor with a more comprehensive understanding of the overarching traffic dynamics. This architectural choice proves to be more effective than the tightly-coupled, end-to-end feature extraction and prediction mechanisms common in other Transformer variants, especially for mitigating error accumulation in long-horizon forecasting. The consistent outperformance, therefore, is not merely an incremental improvement but a validation of our core design philosophy.

#### 4.2.2. Ablation studies.

To dissect the sources of FE-iTransformer’s superior performance and validate our core design principles, we conducted a series of ablation studies on the more complex Urban dataset. These studies are designed to first quantify the overall contribution of the Feature Enhancement Module and then to analyze the necessity of its individual components.

1. Effectiveness of the Feature Enhancement Module (FEM)

To directly measure the impact of our primary innovation, we compare the full FE-iTransformer model against its pure iTransformer backbone. The results of this critical comparison are summarized in Table 3.

**Table 3:** Ablation Study: Impact of the Feature Enhancement Module (FEM).

Model	Metric	30min	60min	90min	120min
iTransformer (Baseline)	MAE	16.37	18.30	19.92	21.58
	RMSE	23.16	25.86	28.25	30.49
FE-iTransformer (Ours)	MAE	16.23	16.64	17.10	17.52
	RMSE	22.92	23.59	24.36	25.03

The data unequivocally demonstrates the significant value added by the FEM. On the Urban dataset, the introduction of the FEM reduces the Mean Absolute Error (MAE) by 0.9%, 9.1%, 14.2%, and a substantial 18.8% for the 30, 60, 90, and 120-minute horizons, respectively. This trend, where the performance gain becomes increasingly pronounced with a longer prediction horizon, strongly suggests that the global context provided by the FEM is highly effective at mitigating the error accumulation problem inherent in long-range forecasting. By enriching the input sequence with a globally-aware context vector, the FEM provides the predictor with a more informative and robust representation, leading to a dramatic improvement in forecasting accuracy.

**Fig 2:**
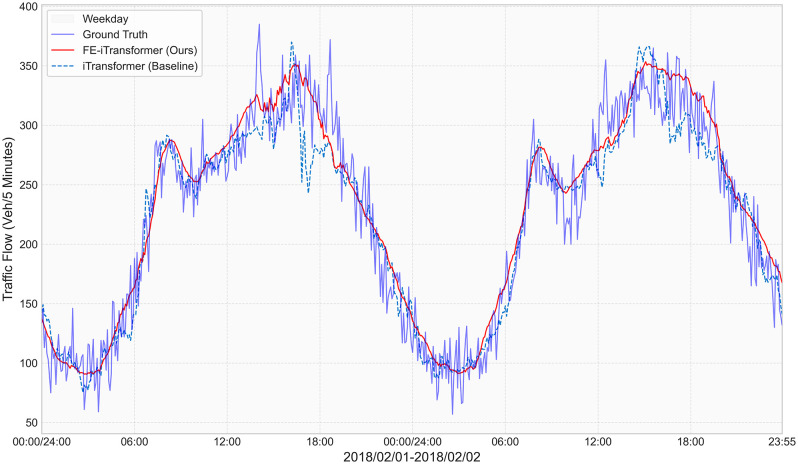
Detailed 48-hour performance comparison on the Urban dataset for the 120-minute prediction horizon. The plotted period (Feb 01–02, 2018) corresponds to workdays (Thursday and Friday).

To provide a qualitative and more intuitive illustration of the performance gains quantified in Table 3, Fig 2 presents a focused visual comparison on the Urban dataset. This visualization plots the 120-minute prediction results of our full FE-iTransformer against its pure iTransformer backbone over a continuous 48-hour period, offering insights into their behavior within a more complex and dynamic traffic environment.

The visualization compellingly demonstrates the sustained and robust performance improvement provided by the Feature Enhancement Module. Throughout the two full daily cycles, which are characterized by less regular patterns compared to highway traffic, our model (solid line) tracks the ground truth with significantly higher fidelity. The baseline iTransformer (dashed line), lacking the global context provided by the FEM, exhibits more pronounced deviations and struggles to capture the correct magnitude and timing of the traffic peaks.

This visual evidence strongly corroborates our quantitative findings on the Urban dataset, validating that our feature enhancement paradigm is crucial for empowering the model to achieve reliable and accurate predictions, especially over extended, multi-cycle forecasting horizons in complex urban scenarios.

1. Necessity of Individual FEM Components

To assess the contribution of each branch in the FEM and validate its multi-branch architecture, we conducted an ablation study in which the three parallel branches were removed one at a time, yielding three model variants with all other components held constant:

w/o Spatio-temporal: Removes the Spatio-temporal Branch.

w/o Periodicity: Removes the Periodicity Branch.

w/o Temporal Context: Removes the Temporal Context Branch.

The performance of these variants is compared against our full model on the Urban dataset, with the results summarized in Table 4.

**Table 4:** Ablation Study of Individual Components within the FEM.

Model Variant	Metric	30min	60min	90min	120min
FE-iTransformer (Full Model)	MAE	**16.23**	**16.64**	**17.10**	**17.52**
	RMSE	**22.92**	**23.59**	**24.36**	**25.03**
w/o Spatio-temporal	MAE	18.25	18.48	18.34	18.97
	RMSE	26.28	26.62	26.54	27.51
w/o Periodicity	MAE	26.83	25.79	25.38	25.94
	RMSE	38.89	38.02	37.66	37.89
w/o Temporal Context	MAE	55.26	58.25	62.06	67.02
	RMSE	67.11	71.92	77.74	84.42

The results, summarized in Table 4, reveal the distinct and complementary roles of each branch within the Feature Enhancement Module. A quantitative analysis of the performance degradation upon the removal of each component confirms their respective and consistent contributions to the model’s overall forecasting accuracy on the Urban dataset.

The removal of the Temporal Context Branch (w/o Temporal Context) leads to the most dramatic performance degradation across all horizons. At the 30-minute horizon, the MAE increases dramatically from 16.23 to 55.26, a surge of approximately 240%. This substantial increase in error underscores the critical importance of explicit timestamp information for the model to accurately anchor its predictions in a specific temporal context.

Similarly, removing the Periodicity Branch (w/o Periodicity) also results in a severe decline in performance. The MAE at the 30-minute horizon rises from 16.23 to 26.83, an increase of about 65%. This finding highlights the necessity of modeling historical cyclical patterns (daily and weekly) to establish a robust baseline for forecasting, especially in the more complex and pattern-driven urban environment.

Finally, the exclusion of the Spatio-temporal Branch (w/o Spatio-temporal) leads to a clear and consistent degradation in prediction accuracy across all prediction horizons. For instance, the MAE increases from 16.23 to 18.25 at the 30-minute mark, and from 17.52 to 18.97 at the 120-minute mark. This demonstrates that even in the presence of strong periodic and temporal signals, the localized spatial patterns and immediate temporal dynamics captured by the CNN-LSTM pipeline provide unique and non-redundant information that is essential for achieving the highest level of accuracy.

An interesting and crucial observation from comparing Table 3 and Table 4 is that the performance of our model variants with a single branch removed (e.g., ‘w/o Spatio-temporal’ with MAE 18.25 at 30 min) is not only significantly worse than the full model (MAE 16.23) but is also inferior to the vanilla iTransformer baseline (MAE 16.37). This phenomenon is not a model flaw but rather a testament to the synergistic nature of our Feature Enhancement Module. We attribute this systematic degradation to the “Latent Distribution Distortion” caused by feature decoupling. In the proposed framework, the FEM branches are optimized jointly to capture complementary components of the traffic signal: the Periodicity Branch explicitly models regular recurring patterns (e.g., daily/weekly cycles), while the Spatio-temporal Branch targets complex local dynamics from the raw input. Consequently, the branches become specialized and interdependent. When a single branch is removed, the resulting global context vector becomes a distorted representation covering only a partial information spectrum (e.g., regular patterns without dynamic context, or dynamics without periodic reference).

Crucially, this distorted feature vector is structurally inferior to the raw input. The vanilla iTransformer backbone, while lacking explicit enhancement, processes the raw sequence which naturally preserves the complete signal integrity (containing both regularities and variations). In contrast, the partial FEM injects a structurally incomplete representation that acts as out-of-distribution noise to the predictor. This forces the backbone to map a skewed feature space to the ground truth, resulting in higher systematic errors than simply learning from the raw, undistorted data.

### 4.3. Validation on a large-scale benchmark

While the ablation studies in the previous section rigorously validated our framework’s internal mechanisms, a potential concern is its scalability and performance on larger, more complex transportation networks. To address this, we conducted a targeted validation experiment on the large-scale PEMS08 public benchmark, which features 170 sensors and significantly more intricate spatio-temporal dynamics. We focus our analysis on the most challenging 120-minute long-horizon forecasting task, as this is where a model’s robustness and ability to mitigate error accumulation are most critically tested.

The performance comparison on the PEMS08 dataset is presented in Table 5. The results unequivocally demonstrate both the successful scalability and the state-of-the-art performance of our proposed framework.

**Table 5:** Performance Comparison on the PEMS08 Dataset for the 120-min Horizon.

Model	MAE	RMSE	MAPE
LSTM	31.84	46.57	20.44%
SVR	31.79	46.82	20.71%
DLinear	27.22	39.98	16.40%
Transformer	27.18	39.62	16.65%
iTransformer	29.68	44.27	18.77%
Autoformer	28.57	42.43	16.27%
PatchTST	29.98	44.16	19,01%
FE-iTransformer	**24.02**	**38.53**	**13.79%**

First, a vertical comparison with its backbone reveals the sustained effectiveness of our core paradigm in a large-scale setting. Even in this complex, high-dimensional environment, FE-iTransformer (MAE 24.02) achieves a remarkable 19.1% reduction in Mean Absolute Error compared to the vanilla iTransformer (MAE 29.68). This provides compelling evidence that the global context vector distilled by the FEM is a robust and scalable mechanism, capable of significantly enhancing the underlying predictor’s capabilities even when faced with intricate, large-scale spatio-temporal dynamics.

Second, a horizontal comparison across different model families highlights the architectural advantages of our approach. We observe that while simple linear models like DLinear show surprisingly competitive performance, they likely benefit from mitigating overfitting on long-horizon tasks but may lack the capacity to capture complex non-linearities. On the other hand, sophisticated Transformer variants such as Autoformer and PatchTST do not exhibit a clear advantage over the vanilla Transformer or even iTransformer in this specific task, with their MAE scores clustering in the 28–30 range. This suggests that their specialized attention mechanisms or patching strategies, while effective in other domains, may not be optimally suited for capturing the unique, multi-faceted dependencies of traffic flow data at this scale.

In stark contrast, our FE-iTransformer establishes a new, significantly lower error benchmark (MAE 24.02). This substantial performance leap indicates that the key to advancing long-horizon traffic forecasting may not lie solely in refining the self-attention mechanism itself, but rather in how the model is supplied with rich, pre-distilled, domain-specific information. Our “extract-and-enhance” philosophy, by decoupling the complex task of multi-faceted feature extraction from the sequence-to-sequence prediction, proves to be a more effective and robust strategy. It empowers a powerful predictor like iTransformer to focus on its core strength—modeling temporal dependencies—while being continuously informed by a comprehensive global context, ultimately leading to a new level of prediction accuracy.

**Fig 3:**
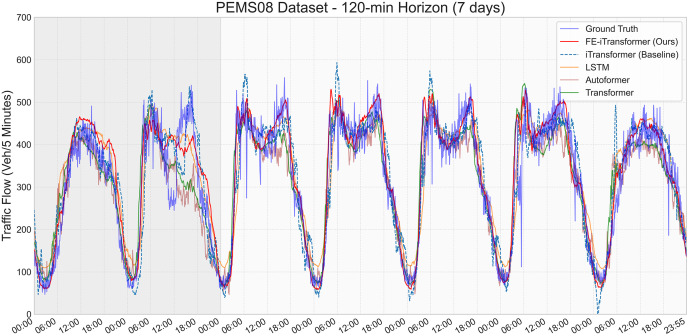
Multi-model prediction performance on the PEMS08 dataset for the 120-minute horizon over a 7-day period. The gray shaded area indicates the weekend (Aug 20–21), and the white area represents workdays.

To provide a more intuitive understanding of our model’s predictive behavior, Fig 3 visualizes the 120-minute prediction results from FE-iTransformer and several key baselines against the ground truth over a 7-day period.

The visualization compellingly illustrates our model’s superior fitting capability. The prediction curve of FE-iTransformer (solid red line) consistently aligns more closely with the ground truth (solid blue line) compared to all other methods. This is particularly evident during traffic peaks and troughs, where many baseline models—including its own backbone iTransformer (dashed blue line) and other competitive models like Autoformer (brown line)—exhibit significant lag, overshooting, or under-prediction.

This qualitative evidence, combined with the quantitative results, strongly suggests that the superior performance stems from the rich, multi-faceted global context provided by the FEM. This allows the FE-iTransformer to maintain a much more accurate and stable trend, demonstrating its robust capability in long-horizon forecasting on complex, large-scale networks. This supplementary experiment, therefore, provides strong evidence that our “extract-and-enhance” paradigm is not only effective in controlled settings but also a robust and scalable solution for real-world scenarios.

### 4.4. Visualization of prediction results

To provide a qualitative and more intuitive assessment of our model’s performance on the primary datasets, this section visualizes the prediction results of FE-iTransformer against the ground truth and key baseline methods. These visualizations serve as a visual counterpart to the quantitative results presented in Section 4.2, offering insights into the models’ dynamic behavior.

**Fig 4:**
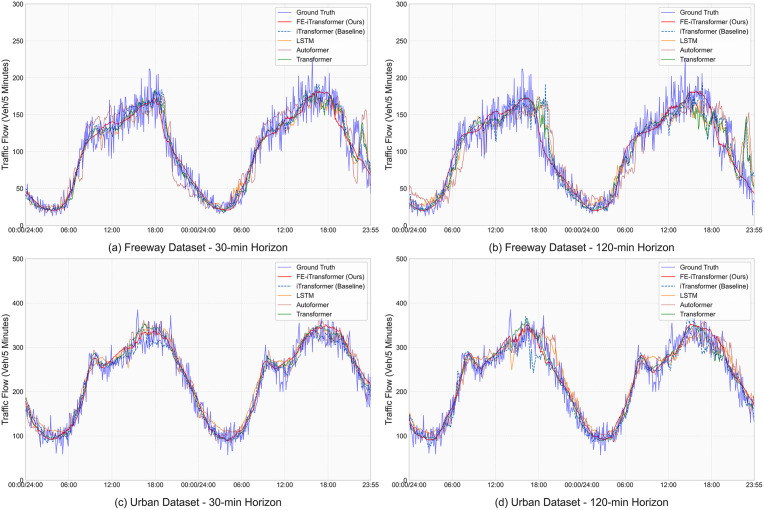
Visualization of multi-model prediction performance. The plots compare FE-iTransformer and baselines on the Freeway (top) and Urban (bottom) datasets for 30-minute (left) and 120-minute (right) horizons. The data spans a 48-hour workday period (Feb 01–02, 2018).

Fig 4 presents a multi-faceted comparison across both the Freeway and Urban datasets at short-term (30-min) and long-term (120-min) prediction horizons. In each subplot, we plot the predictions from our FE-iTransformer, its backbone iTransformer, and other representative baselines against the ground truth curve for a 48-hour period starting from Jan 29, 2018. As can be observed, our model’s predictions (solid red line) consistently align more closely with the ground truth (solid blue line) compared to the other methods. The performance gap is particularly evident in the 120-minute horizon plots (right column), where many baseline models exhibit significant lag or overshooting. In contrast, the FE-iTransformer, benefiting from the global context provided by the FEM, maintains a much more accurate and stable trend, demonstrating its superior capability in long-horizon forecasting. A similar, even more pronounced, superior fitting capability on the large-scale PEMS08 dataset has already been demonstrated and analyzed in Section 4.3.

### 4.5. Computational efficiency and deployment analysis

To evaluate the operational cost of the proposed two-stage architecture, we conducted a quantitative comparison between the FE-iTransformer and its backbone on the PEMS08 dataset. Table 6 presents the efficiency metrics measured with a batch size of 32. The experiments were conducted on a standard NVIDIA GeForce MX350 Laptop GPU.

**Table 6:** Computational efficiency comparison on PEMS08 (170 Nodes).

Model	Parameters (M)	FLOPs (G)	Peak Memory (MB)	Inference Latency (ms/batch)
**iTransformer**	9.51	25.85	114.10	73.03
**FE-iTransformer**	10.06	29.12	124.41	102.54
**Increase Ratio**	+5.8%	+12.6%	+9.0%	+40.4%

As shown, the Feature Enhancement Module (FEM) introduces a slight increase in parameters (+5.8%) and a moderate increase in theoretical FLOPs (+12.6%), indicating that the module is relatively lightweight. We observe a notable increase in inference latency (+40.4%), rising from 73.03 ms to 102.54 ms per batch. This is primarily attributed to the sequential processing required by the recurrent components in the FEM, which restricts the parallel efficiency typical of Transformer architectures.

While this architectural design leads to increased latency, it represents a strategic trade-off between efficiency and robustness. The Spatio-temporal Branch accounts for a significant portion of this latency, its complexity is justified by its specific functional role, which differs fundamentally from the Periodicity Branch. Although ablation studies (Table 4) suggest that removing this branch causes a smaller rise in MAE compared to removing periodic features, this metric primarily reflects the dominance of repetitive patterns in traffic data. The Periodicity Branch captures these easy-to-learn base patterns. In contrast, the Spatio-temporal Branch is tasked with the far more challenging objective: capturing high-frequency, non-recurrent dynamics and sudden traffic shifts. Simpler alternatives, such as standard CNN-GRU pipelines, often lack the global context awareness to distinguish these anomalies from noise. Therefore, the additional computational cost is a necessary investment to ensure model robustness in complex, dynamic scenarios.

Deployment Feasibility: Despite the increased latency, we believe the FE-iTransformer remains feasible for practical Intelligent Transportation Systems (ITS):

1. Time Constraints: The inference time of ~102 ms is well within the typical 5-minute (300,000 ms) data collection interval of traffic systems, leaving sufficient margin for real-time operations.
2. Resource Usage: The peak memory consumption (~124 MB) remains manageable for standard industrial edge devices.
3. Cost-Benefit Balance: Considering the 19.1% reduction in MAE for long-horizon forecasting, we consider the additional computational overhead to be an acceptable trade-off for high-stakes traffic management tasks.

### 4.6. Applicability scope and methodological positioning

It is worth discussing the positioning of FE-iTransformer relative to Graph Neural Networks (GNNs), which represent another dominant paradigm in traffic forecasting. GNNs (e.g., STGCN, DCRNN) achieve high performance by explicitly leveraging pre-defined adjacency matrices to model spatial topology. In scenarios where high-quality, static road graphs are readily available, GNNs remain a powerful choice.

However, the design of FE-iTransformer targets a different, yet equally critical practical constraint: the “No-Graph” or “Weak-Graph” setting. In many real-world deployments—such as ad-hoc sensor networks or privacy-preserved data sharing—topological information is often missing or unreliable. Under these constraints, graph-dependent models may suffer from performance degradation or become inapplicable.

Therefore, our experimental design focuses on comparing FE-iTransformer against graph-agnostic baselines (e.g., iTransformer, PatchTST). This ensures a rigorous evaluation where all models operate under identical information conditions (i.e., time series data only). The results demonstrate that FE-iTransformer effectively compensates for the lack of explicit topological priors by distilling implicit spatio-temporal dynamics via the FEM. This positions the proposed framework not as a direct competitor to GNNs in graph-rich environments, but as a robust, deployment-ready alternative for scenarios where maintaining a reliable graph structure is costly or infeasible.

## 5. Conclusion

In this paper, we proposed a novel two-stage framework for high-accuracy, long-horizon traffic flow forecasting, named the Feature-Enhanced iTransformer (FE-iTransformer). Motivated by the persistent challenges in capturing complex spatio-temporal dependencies without relying on predefined graph structures, our work introduces an “extract-and-enhance” paradigm. This paradigm first employs a comprehensive Feature Enhancement Module (FEM) to distill a potent global context vector from multi-source information, including spatio-temporal dynamics, periodic patterns, and temporal context. Subsequently, an innovative per-step feature enhancement mechanism leverages this global context to enrich the original input sequence, which is then processed by a powerful iTransformer backbone for final prediction.

The effectiveness of the proposed FE-iTransformer was rigorously validated through a multi-faceted experimental evaluation. First, in-depth experiments on our primary datasets (Freeway and Urban) established the model’s superior performance over a wide range of state-of-the-art baselines and, through detailed ablation studies, provided compelling evidence for the efficacy of our two-stage design. The introduction of the FEM was shown to significantly improve the performance of the pure iTransformer backbone, with the performance gain becoming increasingly pronounced in the most challenging long-horizon forecasting tasks. Second, supplementary experiments on the large-scale PEMS08 benchmark further confirmed the framework’s robustness and scalability, where it again achieved state-of-the-art results, reducing the MAE by 19.1% in the 120-minute horizon. These key findings strongly validate the superiority of decoupling the complex feature extraction process from the final sequence prediction task.

Despite the promising results, this study identifies opportunities for future improvement. First, the current model relies solely on historical traffic observations; future iterations will extend the Feature Enhancement Module (FEM) to incorporate exogenous signals such as weather conditions and traffic incidents. Second, regarding computational efficiency, the two-stage architecture introduces additional overhead compared to single-stage baselines. While our analysis confirms that the inference latency remains well within real-time deployment limits (as discussed in Section 4.5), the sequential processing in the FEM limits parallelization efficiency. Future work will therefore explore lightweight alternatives, such as adaptive gating mechanisms or efficient attention variants, to further optimize the accuracy-efficiency trade-off for strictly resource-constrained edge devices.
